# Correlation of Oxidative and Antioxidative Processes in the Blood of Patients with Cervical Spinal Cord Injury

**DOI:** 10.1155/2016/6094631

**Published:** 2016-01-05

**Authors:** Bartosz Woźniak, Alina Woźniak, Celestyna Mila-Kierzenkowska, Heliodor Adam Kasprzak

**Affiliations:** ^1^Department of Neurosurgery, Stanisław Staszic Specialist Hospital, Rydygiera 1, 64-920 Piła, Poland; ^2^The Chair of Medical Biology, Collegium Medicum, Nicolaus Copernicus University, Karłowicza 24, 85-092 Bydgoszcz, Poland

## Abstract

The effect of cervical spinal cord injury (CSCI) on oxidative stress parameters was assessed. The study was conducted in 42 patients with CSCI (studied group), 15 patients with cerebral concussion, without CSCI (Control II), and 30 healthy volunteers (Control I). Blood was taken from the basilic vein: before and seven days after the spinal cord decompression surgery (mean time from CSCI to surgery: 8 hours) in the studied group and once in the controls. Thiobarbituric acid reactive substances (TBARS) and conjugated dienes (CD) concentrations, and glutathione peroxidase (GPx), catalase (CAT), and creatine kinase (CK) activities before the surgery were higher in the studied group than in the controls. Reduced glutathione concentration was similar in all groups. Superoxide dismutase (SOD) in the studied group was 16% lower (*P* ≤ 0.001) than in Control I. Lipid peroxidation products, and GPx and CAT activities in erythrocytes seven days after the surgery were lower (*P* ≤ 0.001), while SOD was 25% higher (*P* ≤ 0.001) than before the surgery. CK in blood plasma after the surgery was 34% lower (*P* ≤ 0.001) than before it. CSCI is accompanied by oxidative stress. Surgical and pharmacological treatment helps to restore the oxidant-antioxidant balance.

## 1. Introduction

Traumatic injury to the spinal cord can produce a neurological injury with many physiological, psychological, and social consequences. It is a devastating event that can result in the loss of voluntary motor and sensory function below the level of the injury [1]. Motor-vehicle accidents, sports and recreational activities, accidents at work, and falls at home are identified as the main causes of spinal cord injuries (SCIs) [1, 2]. The use of different kinds of protective equipment, such as protective helmet or seat belt, and observing speed limits result in a decrease in both the mortality and the frequency of occurrence of serious disability. However, lifelong disabilities following an SCI remain one of the most complex and unresolved problems for neuroscientists. Preliminary surgical and pharmacological treatment and early rehabilitation are considered the principal factor influencing the final outcome of the trauma. Many years of clinical investigations have given rise to a model of surgical and pharmacological treatment of patients with cervical spine cord injury (CSCI), but it is still necessary to conduct extensive research of the secondary injury, that is, the outcome of biochemical disturbances in the injured spinal cord tissue.

Studies by many authors have confirmed the existence of several secondary biochemical processes that lead to the spreading of postinjury damage to the CNS. These biochemical processes may result in an increasing neurological deficit and even death [3, 4]. The secondary injury cascade follows the initial injury and appears to be mediated by cellular and molecular processes that act through a complex mechanism [5].

The increased generation of reactive oxygen species (ROS) during an SCI appears to play an important role in neuronal cell death and neurological dysfunction [3, 6]. ROS are molecules containing oxygen but with higher reactivity than the ground state of oxygen. Among them are free radicals, for example, superoxide (O_2_^•−^) and hydroxyl radical (^•^OH), and nonradicals such as hydrogen peroxide (H_2_O_2_). The high reactivity of these oxygen species is the reason for which they can easily attack critical cellular components, including nucleic acids, proteins, and phospholipids, and thus promote neuronal degeneration [3]. Lipid peroxidation is the major form of radical-induced oxidative damage that occurs in injured nervous tissue [6]. The products of this process include, for example, conjugated dienes (CD) and aldehydes, such as malondialdehyde (MDA) [7] and 4-hydroxynonenal (4-HNE) [8, 9]. The attack of ROS on polyunsaturated fatty acids in neuronal cell membranes may trigger peroxidative degeneration that destroys membrane phospholipids following CNS injury [6]. The release of free fatty acids from neuronal membranes is one of the first processes in the sequence of events that accompany the primary injury to the spinal cord neurons [10].

Organisms have developed a complex system of antioxidant defence mechanisms to limit overaccumulation of ROS in the body [11]. One of the defence mechanisms is based on antioxidant enzymes and the other one is based on nonenzymatic scavengers of free radicals. The most important mechanism is the enzymatic antioxidant barrier. The crucial enzymes involved in this process are superoxide dismutase (SOD), glutathione peroxidase (GPx), thioredoxin reductase (TR), and catalase (CAT). SOD eliminates superoxide by catalysing the reaction of dismutation [12]. CAT and GPx catalyse the disintegration of hydrogen peroxide and thus protect the cell against the formation of the most reactive hydroxyl radical [12]. Nonenzymatic antioxidants include ascorbic acid (vitamin C), reduced glutathione (GSH), *α*-tocopherol (vitamin E), flavonoids, carotenoids, selenium, and melatonin [11]. GSH, the main antioxidant in CNS, acts through a nonenzymatic mechanism directly on ROS, mainly superoxide radicals, hydroxyl radicals, nitric oxide, and carbon radicals, leading to their removal [13].

It is well known that an SCI is accompanied by damage not only to the nervous tissue-cerebrospinal fluid barrier, but also to the nervous tissue-blood barrier. For this reason, it seems probable that pro- and antioxidant processes taking place in the central nervous system can be reflected in the composition of both the cerebrospinal fluid and blood.

The presented study examined the effect of CSCI and the subsequent therapeutic procedures on the concentration of lipid peroxidation products (thiobarbituric acid reactive substance (TBARS) and CD) in blood plasma and erythrocytes and on the activities of antioxidant enzymes (SOD, CAT, and GPx) in erythrocytes. Appropriate measurements were made before the surgery and one week after it. Additionally, the concentration of GSH and the activity of creatine kinase (CK), a muscle injury marker, were measured in the study.

## 2. Materials and Methods

### 2.1. Subjects

The study involved 42 persons (29 men and 13 women, mean age 38.3 ± 5.5 years) with CSCI (studied group), 15 persons (12 men and 3 women, mean age 39.3 ± 6.7 years) with cerebral concussion (Control II), and 30 healthy volunteers (25 men and 5 women, mean age 33.3 ± 7.5 years) (Control I). All patients were admitted to the division of neurosurgery. Subjects with other health problems involving a proven oxidant-antioxidant imbalance were excluded from the study. None of the subjects received medication or vitamin supplementation known to interfere with the antioxidant system. The study was approved by the Bioethics Committee at Collegium Medicum in Bydgoszcz, Nicolaus Copernicus University in Toruń, Poland. All subjects provided their written informed consent.

Patients with CSCI were transported to the hospital in a fully immobilised position with an orthopaedic collar. Diagnosis was based on neurological and radiological examinations. The radiological examination included X-ray, computed tomography (CT), and magnetic resonance imaging (MRI).

The cerebral concussion group (Control II) included patients with mild traumatic brain injury (no loss of consciousness, transient confusion, and postconcussion symptoms clear in less than 15 min), involving a transient disturbance of the spinal cord function without SCI.

Neurological examinations of the patients with SCI were performed three times: at the time of admission to the hospital, after the cranial retraction, and seven days after the surgery. The patients with brain concussion were subjected to neurological evaluation only once: at the time of admission to the hospital. The neurological assessment was based on the American Spinal Injury Association (ASIA) Impairment Scale (Table 1). The initial neurological deficit was estimated 24 hours after the trauma.

The assessment of patients with multiple injuries, derived from both the CSCI group (studied group) and the brain concussion group (Control II), was performed at the time of admission to the hospital. The assessment was based on the Injury Severity Score (ISS) (Table 2).

The spinal cord decompression surgery was an emergency procedure performed within less than 12 hours after CSCI. Mean time between CSCI and the decompression surgery was 8 hours.

Before hospitalisation, each patient received an intravenous injection of methylprednisolone at a high dose of 30 mg/kg bw [14, 15]. The following dose of 8.5 mg methylprednisolone/kg bw was administered by means of infusion during the subsequent 23 hours. On the day after the surgery, methylprednisolone was supplied orally at gradually decreasing doses. The steroid therapy was ceased by the fourth day of hospitalisation. During the steroid treatment, the patients from the studied group received ranitidine at a dose of 0.3 g BID to avoid stomach erosion. Respiratory efficiency was monitored using standard gasometry. The patients with abnormal gasometric readings were excluded from the studied group. Immediately before and after the surgery, 1.5 g cefuroxime was intravenously administered to the patients. The cefuroxime therapy was continued over the following four days. Before the surgery, all patients were subjected to cervical traction using the Crutchfield method with the retraction power of 10% bw. The retraction was performed under radiological control to verify the reposition of vertebrae. The neurosurgical treatment induced decompression of the neural and vascular elements of the spinal cord. Stabilisation of the spinal column via anterior spondylodesis was performed as well, using the Cloward or Smith-Robinson methods. After the radiological control of cervical spine stability, all patients were subjected to active and passive rehabilitation exercises of the paralysed limb three times a day for 2 hours.

### 2.2. Sample Preparation

The study material, venous blood, was taken in a fasting state from the basilic vein of the studied group members at two time points: before the surgery and seven days after it. Blood samples from the control groups were taken once, following the same procedure as in the patients from the studied group. The blood samples were treated with 50 *μ*L of heparin. Blood plasma was obtained by the centrifugation of blood at 3000 ×g, followed by decantation of the liquid above the cell sediment. The erythrocyte mass, obtained from a thin layer of the erythrocyte sediment, was washed three times with phosphate buffer saline (PBS) to obtain an erythrocyte suspension with a 50% dispersion coefficient.

### 2.3. Assay of Lipid Peroxidation Products in Blood Plasma and Erythrocytes

The estimation of TBARS concentration in blood plasma and erythrocytes was performed according to the method by Buege and Aust [16] as modified by Esterbauer and Cheeseman [17]. The method involves formation of a coloured complex between lipid peroxidation products and thiobarbituric acid at a temperature of 100°C in an acidic environment. The maximum absorption of that complex occurs at a wavelength of 532 nm. The principal member of TBARS is malondialdehyde (MDA); therefore the concentration of TBARS is expressed in nmol MDA/mL in blood plasma and in nmol MDA/g Hb in erythrocytes.

The level of CD was determined following Sergent et al. [18]. This method involves measuring of the absorbance peak characteristic for dienes at a wavelength of *λ* = 233 nm. The level of CD is expressed in absorbance units per millilitre of blood plasma and per gram of haemoglobin, respectively (Abs./mL and Abs./g Hb).

### 2.4. Assay of Reduced Glutathione Concentration and Antioxidant Enzyme Activity in Erythrocytes

GSH concentration in erythrocytes was assessed using the Calbiochem (USA) commercial kit. The kit is based on a chemical reaction that occurs in two steps. The first step leads to the formation of substitution products (thioethers) between a proprietary chromogenic reagent and all mercaptans (RSH) present in the sample. The second step is a *β*-elimination reaction that takes place in alkaline conditions. This reaction is mediated by 30% NaOH which specifically transforms the substitution product obtained with GSH into a chromophoric thione with the maximal absorbance at 400 nm [19]. GSH concentration is expressed in *μ*mol.

SOD activity was determined according to the Misra and Fridovich [20] method based on the inhibition of autooxidation of adrenaline to adrenochrome by SOD in an alkaline environment. The unit of SOD activity is the quantity of the enzyme that inhibits the reaction by 50% at a maximum increase in absorption of 0.025 units/min on a rectilinear section of adrenochrome formation. SOD activity was expressed as U/g Hb.

The Beers and Sizer [21] method was used to determine CAT activity. This method is based on the measurement of a decrease in the absorbance of hydrogen peroxide which is decomposed by catalase, measured at a wavelength of 240 nm. CAT activity was expressed as IU/g Hb.

GPx activity was assayed according to the Paglia and Valentine [22] method. This method is based on the measurement of changes in absorbance at a wavelength of 340 nm caused by the oxidation of reduced nicotinamide adenine dinucleotide phosphate (NADPH). NADPH is a coenzyme in the reduction of glutathione disulphide. The obtained oxidized glutathione is a product of the reaction catalysed by GPx. GPx activity was expressed as U/g Hb.

### 2.5. Assay of Creatine Kinase Activity in Blood Plasma

CK activity in blood plasma was assessed using a commercial kit from POCH S.A., Gliwice (Poland), based on the kinetic method by Oliver [23], as modified by Rosalki [24] and Szasz et al. [25]. This method is based on the measurement of absorbance increase at a wavelength of 400 nm caused by the reduction of NADP to NADPH. In this method, creatine kinase catalyses the formation of ATP from ADP and creatine phosphate. In the presence of hexokinase, ATP converts glucose to glucose-6-phosphate. Glucose-6-phosphate is oxidized to 6-phosphogluconate with a simultaneous reduction of NADP to NADPH. CK activity is expressed in IU/l.

### 2.6. Statistical Analysis

The results are expressed as means ± standard deviation (SD). Statistical analysis was conducted using the ANOVA test with post hoc analysis (Tukey's range test) (*STATISTICA v.* 9.1). A hypothesis of the equality of two means was tested. The conformity with the normal distribution was determined on the basis of the Shapiro-Wilk test. The equality of variances was assessed using Levene's test. Differences at a significance level *P* ≤ 0.05 were presumed as statistically significant. Dependencies between the analysed parameters were assessed using correlation matrices. A statistical hypothesis of the significance of the correlation coefficients (*r*) was tested.

## 3. Results

### 3.1. Radiological Characterization of the CSCI Patients

The radiological examinations (X-ray and CT) revealed significant changes in the CSCI patients. In two patients, fracture of the C3 vertebra with anterior dislocation of the C3 and C4 vertebrae was observed. Fracture of the C4 and C5 vertebrae with anterior dislocation of the C4 and C5 vertebrae was found in 5 patients, while fracture of the C5 pedicle was observed in 25 patients. Six patients had a simultaneous fracture of the C6 pedicle. Dislocation of the C5 and C6 vertebrae was noted in 8 patients, while dislocation of the C6 to C7 vertebrae was found only in 2 cases.

Spinal cord compression, caused by vertebral disc and/or by fractured and dislocated bone mass, was registered in MRI. The extent of compression was less than 2 millimetres in 8 patients, more than 2 but less than 4 millimetres in 20 patients, and 4 millimetres or more in 9 patients.

### 3.2. Evaluation of Oxidative Stress Parameters

The concentration of lipid peroxidation products in the studied group before the surgery was higher than in Controls I and II in a statistically significant manner (Table 3). TBARS concentration in the blood plasma of the CSCI patients before the surgery was more than 2 times higher (*P* ≤ 0.05) than in Control I and 44% higher (*P* ≤ 0.001) than in Control II. In erythrocytes, TBARS concentration was 84% (*P* ≤ 0.001) and 36% (*P* ≤ 0.001) higher, respectively. CD concentration in the blood plasma of the CSCI patients before the surgery was more than 2 times higher (*P* ≤ 0.001) than in Control I and 38% higher (*P* ≤ 0.001) than in Control II. In erythrocytes, CD concentration was 79% (*P* ≤ 0.001) and 30% (*P* ≤ 0.01) higher, respectively. CAT, GPx, and CK activities in the studied group before the surgery were also statistically significantly higher than in Control I, differing by 68% (*P* ≤ 0.001), 54% (*P* ≤ 0.001), and 102% (*P* ≤ 0.001), respectively. CAT activity in the erythrocytes of the studied group before the surgery was approx. 18% higher (*P* ≤ 0.01) than in the patients with cerebral concussion (Control II). The activity of SOD in the erythrocytes of the patients from the studied group before the surgery was approx. 16% (*P* ≤ 0.001) lower than in Control I and approx. 6% (*P* ≥ 0.05) lower than in Control II. GSH concentration was similar in all groups.

Comparing TBARS concentrations in the blood plasma and erythrocytes of the patients from the studied group 7 days after the surgery with the results of both control groups, levels higher in a statistically significant manner were obtained only in the comparison with healthy volunteers, differing by 46% (*P* ≤ 0.001) and 31% (*P* ≤ 0.001), respectively. CD concentration in the blood plasma of the CSCI patients 7 days after the surgery was 29% higher (*P* ≤ 0.001) than in Control I and 21% lower (*P* ≤ 0.05) than in Control II. CD concentration in the erythrocytes of the CSCI patients 7 days after the surgery was 10% higher (*P* ≤ 0.05) than in Control I and 20% lower (*P* ≤ 0.01) than in Control II. SOD activity in the erythrocytes of the CSCI patients 7 days after the surgery did not differ significantly in comparison with that measured in the healthy volunteers, while it was 17% higher (*P* ≤ 0.01) than in the patients with cerebral concussion. CAT activity at that time point of the study was 19% higher (*P* ≤ 0.001) than in the healthy volunteers from Control I and 17% lower (*P* ≤ 0.001) than in Control II. GPx activity in the erythrocytes of the patients from the studied group 7 days after the surgery did not differ significantly in comparison with that measured in Control I but was 32% lower (*P* ≤ 0.001) than in Control II. GSH concentration determined in the studied group 7 days after the surgery did not differ in a statistically significant manner from that determined in both control groups, whereas CK activity was 34% (*P* ≤ 0.001) higher than in Control I and 32% lower (*P* ≤ 0.001) than in Control II.

When comparing the data obtained before the surgery and 7 days after the surgery in the CSCI patients, it was found that TBARS concentration was approx. 34% (*P* ≤ 0.001) lower in blood plasma and 28% (*P* ≤ 0.001) lower in erythrocytes on the seventh day after the surgery.

The levels of CD in both blood plasma and erythrocytes on the seventh day after the surgery were also lower, approx. 43% (*P* ≤ 0.001) and 39% (*P* ≤ 0.001), respectively, than before the surgery.

SOD activity was approx. 25% higher on the seventh day after the surgery as compared with that obtained in the presurgery examination (*P* ≤ 0.001), while CAT and GPx activities were lower (approx. 29%, *P* ≤ 0.001, and 32%, *P* ≤ 0.001, resp.) on the seventh day after the surgery than before it.

The concentration of GSH in the examination after the surgery was unchanged compared to the value before the surgery. CK activity measured 7 days after the surgery was approx. 34% lower compared to that obtained in the presurgery examination (*P* ≤ 0.001).

**Figure 1:**
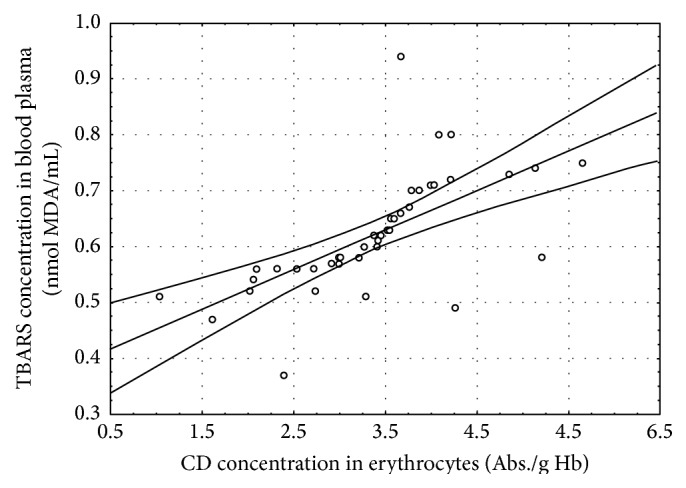
Linear regression (*r* = 0.65, *P* ≤ 0.001) of conjugated dienes (CD) concentration in erythrocytes versus thiobarbituric acid reactive substances (TBARS) concentration in the blood plasma of the patients with cervical spinal cord injury before the surgery.

**Figure 2:**
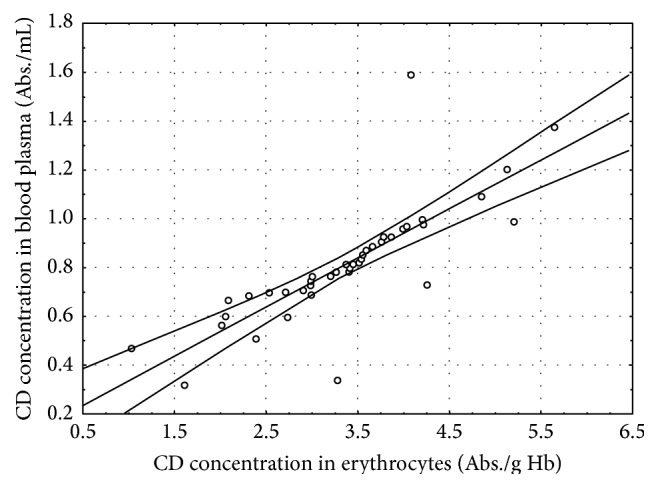
Linear regression (*r* = 0.80, *P* ≤ 0.001) of conjugated dienes (CD) concentration in erythrocytes versus CD concentration in the blood plasma of the patients with cervical spinal cord injury before the surgery.

**Figure 3:**
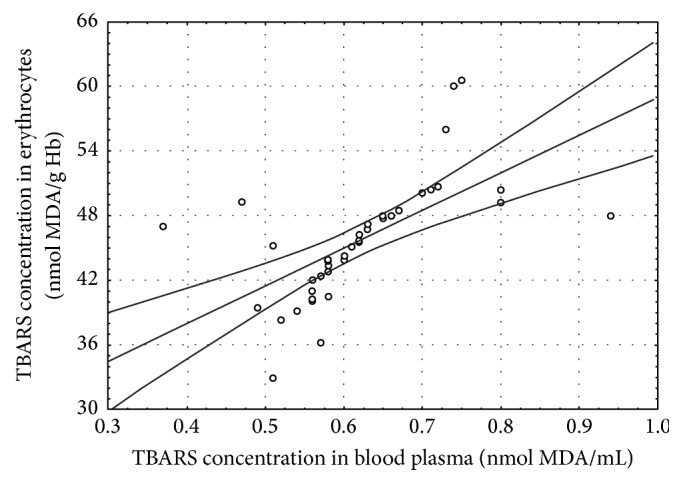
Linear regression (*r* = 0.64, *P* ≤ 0.001) of thiobarbituric acid reactive substances (TBARS) concentration in blood plasma versus TBARS concentration in the erythrocytes of the patients with cervical spinal cord injury before the surgery.

**Figure 4:**
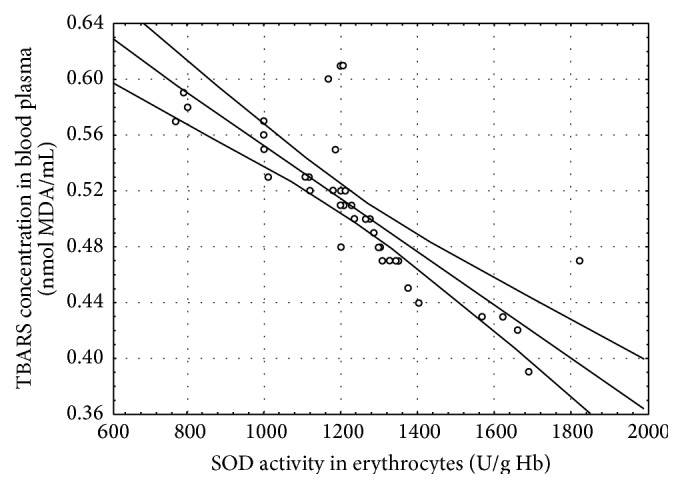
Linear regression (*r* = −0.80, *P* ≤ 0.001) of superoxide dismutase (SOD) activity in erythrocytes versus thiobarbituric acid reactive substances (TBARS) concentration in the blood plasma of the patients with cervical spinal cord injury 7 days after the surgery.

**Figure 5:**
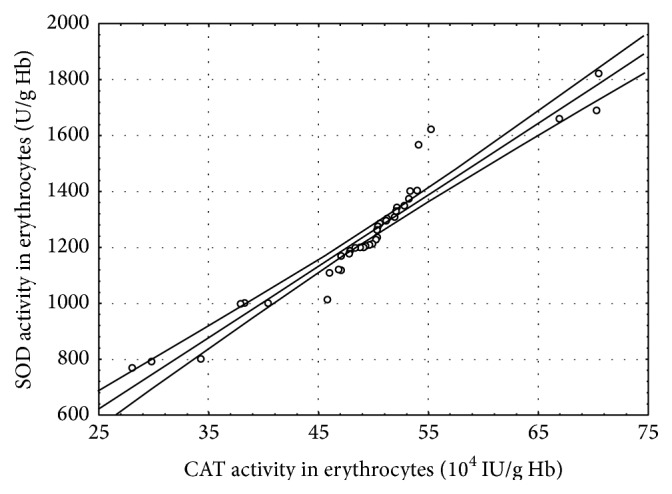
Linear regression (*r* = 0.96, *P* ≤ 0.001) of catalase (CAT) activity versus superoxide dismutase (SOD) activity in the erythrocytes of the patients with cervical spinal cord injury 7 days after the surgery.

In the CSCI patients, several statistically significant correlations between the determined parameters were found at both time points of the study (Table 4, Figures 1–5).

## 4. Discussion

The concentrations of TBARS and CD in the blood of the patients with CSCI before the surgery were higher in a statistically significant manner than in the healthy volunteers (Control I) and in the patients with brain concussion (Control II). The concentrations of these lipid peroxidation products were also higher before the surgery than 7 days after the surgery. Moreover, a positive correlation between the CD levels in erythrocytes and the TBARS concentration in blood plasma before the surgery (*r* = 0.65, *P* ≤ 0.001) was observed (Figure 1). Similar positive correlations between the CD levels in erythrocytes and blood plasma (*r* = 0.80, *P* ≤ 0.001) and between the TBARS concentrations in blood plasma and erythrocytes (*r* = 0.64, *P* ≤ 0.001) were found (Table 4, Figures 2 and 3). The obtained results confirm that damage to the cervical spinal cord is accompanied by increased peroxidation of lipids as a consequence of increased ROS generation. Many authors confirm the existence of increased ROS generation resulting from SCI [3, 8, 26, 27].

There are probably many mechanisms responsible for the increased generation of ROS in the course of SCI. The cascade of reactions of oxygen radical begins with the generation of O_2_^•−^ in response to a rapid increase in intracellular Ca^2+^ concentration directly after mechanical damage to the spinal cord [28]. In solution, the superoxide anion exists in an equilibrium with the hydroperoxyl radical (HO_2_^•^). In tissue acidosis, which accompanies SCI, the balance changes to the benefit of the hydroperoxyl radical which is more reactive than O_2_^•−^, especially against lipids [5]. The superoxide anion reacts with the nitric oxide radical (^•^NO) and forms a highly reactive oxidizing agent, peroxynitrite (ONOO^−^), and a hydroxyl radical as a by-product. Increased generation of the hydroxyl radical also occurs in the Fenton reaction in which ferrous iron (Fe^2+^) is oxidized to form ^•^OH in the presence of H_2_O_2_. Increased concentration of iron ions during SCI is a result of its release from transferrin and ferritin, which occurs when pH decreases, and from haemoglobin, which occurs after a mechanically induced haemorrhage [28]. The hydroxyl radical and other peroxynitrite-derived radicals can initiate the lipid peroxidation process [5].

Springer et al. [8] and Xiong et al. [9] noticed an increased concentration of 4-hydroxynonenal, one of the lipid peroxidation markers, after a spinal cord trauma. Massive release of glutamate has also been observed [8, 29, 30]. This process may be responsible for the excessive influx of calcium ions into the neuron cytoplasm. Disturbances in calcium homoeostasis may lead to the activation of many enzymes, such as calpain, and can also cause disruption of the mitochondrial respiratory chain. Other authors also confirmed an increased calpain activity in animals with an experimental spinal cord injury [31]. The activation of this enzyme leads to protein damage and to the conversion of xanthine dehydrogenase into oxidase. This process may cause increased superoxide generation and lipid peroxidation [32]. Increased lipid peroxidation after CSCI was also confirmed in our previous study in which higher TBARS and CD concentrations in the blood of patients compared to healthy individuals were determined. Reduced antioxidative potential of blood plasma was also observed in these patients [7].

In the presented study, SOD activity was lower before the surgery than 7 days after the surgery. On the contrary, CAT and GPx activities immediately after the trauma (before the surgery) were higher in a statistically significant manner than 7 days after the surgery. The obtained results indicate changes in the functioning of the enzymatic antioxidant barrier in the patients with CSCI. Disturbance in the oxidant-antioxidant balance in subjects with SCI was also confirmed by Bastani et al. [33] who observed lower levels of plasma antioxidants and higher concentration of 8-epiprostaglandin F2*α*, an oxidative stress biomarker, in urine one month after injury, as compared with healthy individuals.

The activity of the enzymes responsible for the decomposition of hydrogen peroxide (CAT and GPx), which is statistically significantly higher after the trauma, may be a proof of the increased H_2_O_2_ generation. Hydrogen peroxide is the only substrate of catalase, while GPx is also capable of eliminating lipid hydroperoxides. Hence, an increase in the enzyme activity may indicate an increase in oxidative stress. Kaynar et al. [34] demonstrated an increase in CAT activity after an experimental injury of the spinal cord. Yet the same authors did not observe any changes in SOD and GPx activities. However, the authors evaluated the activities of the enzymes directly in the injured spinal cord tissue and compared them with the activities assessed in the normal nerve tissue located next to the damaged region. A decrease in the activities of these enzymes, observed in our study 7 days after the surgery, may indicate improved oxygen metabolism and decreased generation of hydrogen peroxide. The activity of SOD immediately after the spinal cord trauma, lower than 7 days after the surgery, indicates that the enzyme is suppressed just after the injury or that the observed increase in lipid peroxidation is caused by forms of ROS other than superoxide (O_2_^•−^), the only substrate of this enzyme. As was previously mentioned, during an injury to the nervous tissue and the following local acidosis, superoxide is probably protonated and converted into hydroperoxyl radical (HO_2_^•^). This change leads to an increase in lipophilicity and also to the loss of substrate affinity to SOD [35]. The surgical decompression of the nervous and vascular structures improves perfusion resulting in better oxygenation, lower acidosis, and, probably, slower O_2_^•−^ protonation. Seven days after the surgery, statistically significant negative correlations between the SOD activity and TBARS concentration in blood plasma (*r* = −0.80, *P* ≤ 0.001) (Figure 4) and erythrocytes (*r* = −0.36, *P* ≤ 0.05), as well as between the SOD activity and CD levels in erythrocytes (*r* = −0.51, *P* ≤ 0.01), were revealed. These correlations may prove that the long-term effect of neurosurgical treatment is a reduction in the oxidative stress caused by the trauma. The main product of most reactions involving the generation of ROS is superoxide [36]. This radical is unable to initiate the lipid peroxidation process, but it is involved in the formation of other ROS, such as the hydroxyl radical or peroxynitrite, which are capable of initiating lipid peroxidation. Therefore, efficient scavenging of superoxide may decrease lipid peroxidation. The key role of SOD in SCIs is indicated by experimental studies in animals. It has been proven that, in rats with SCI, intravenous administration of lecithinized superoxide dismutase improves spinal cord injury-induced motor dysfunction through the suppression of oxidative stress and enhancement of neurotrophic factor production [37].

A strong positive correlation between SOD and CAT activities (*r* = 0.96, *P* ≤ 0.001) 7 days after the surgery was also observed (Figure 5). This fact suggests that it is catalase, not GPx, that plays the key role in the elimination of hydrogen peroxide, a product of dismutation catalysed by SOD. The hypothesis is also confirmed by the statistically insignificant differences in the concentration of GSH, basic substrate of GPx, obtained in the study. The fact that the activity of CK 7 days after the surgery was lower than immediately after the spinal cord injury can be a proof of inhibition of muscle degeneration. The degeneration is a result of weaker efferent impulses due to the trauma to the central nervous system.

## 5. Conclusions

The obtained results confirm that damage to the cervical spinal cord is accompanied by oxidative stress. This is evidenced by the observed changes in the activity of antioxidant enzymes with the concurrently higher concentrations of lipid peroxidation products than in the control groups. The implemented decompression surgery and pharmacological treatment help to restore the oxidant-antioxidant balance.

## Figures and Tables

**Table 1:** Neurological status observed in patients with cervical spinal cord injury (studied group), patients with brain concussion (Control II), and healthy volunteers (Control I).

ASIA grade	Studied group			Control II	Control I
	At the time of admission to the hospital	After the cranial Crutchfield instrumentation	7 days after surgery	At the time of admission to the hospital	During blood sample taking
A	7	5	4	0	0
B	10	10	9	0	0
C	12	11	9	4	0
D	8	10	11	3	0
E	5	6	9	8	30

ASIA: American Spinal Injury Association Impairment Scale (A: no motor or sensory function is preserved in the sacral segments; B: sensory, but not motor, function is preserved below the neurological level and includes the sacral segments; C: motor function is preserved below the neurological level, and more than half of key muscles below the neurological level have muscle grade < 3; D: motor function is preserved below the neurological level, and at least half of key muscles below the neurological level have muscle grade ≥ 3; E: motor and sensory functions are normal).

**Table 2:** The Injury Severity Score (ISS) in patients with cervical spinal cord injury (studied group) and patients with brain concussion (Control II) at the time of admission to the hospital.

ISS	* *Number of patients	
	From the studied group	From Control II
41	1	0
34	1	0
29	5	2
24	3	2
22	6	5
17	6	5
14	6	1
9	14	0

**Table 3:** Concentrations of lipid peroxidation products: thiobarbituric acid reactive substances (TBARS) and conjugated dienes (CD); activities of antioxidant enzymes: superoxide dismutase (SOD), glutathione peroxidase (GPx), and catalase (CAT); concentration of reduced glutathione (GSH), as well as creatine kinase (CK) activity in healthy volunteers (Control I), patients with cerebral concussion (Control II), and patients with injured cervical part of the spinal cord before and 7days after the surgery (studied group).

Parameters	Healthy volunteers (Control I)	Patients with cerebral concussion (Control II)	* *Studied group	
			Before the surgery	7 days after the surgery
TBARS in blood plasma (nmol MDA/mL)	0.28 ± 0.06	0.43 ± 0.04^aaa^	0.62 ± 0.01^abbb^	0.41 ± 0.05^*∗∗∗*aaa^
TBARS in erythrocytes (nmol MDA/g Hb)	24.9 ± 2.6	33.7 ± 3.9^aaa^	45.7 ± 5.7^aaabbb^	32.7 ± 5.5^*∗∗∗*aaa^
CD in blood plasma (Abs./mL)	0.34 ± 0.03	0.56 ± 0.12^aaa^	0.77 ± 0.24^aaabbb^	0.44 ± 0.15^*∗∗∗*aaab^
CD in erythrocytes (Abs./g Hb)	1.94 ± 0.26	2.67 ± 0.67^aaa^	3.48 ± 0.94^aaabb^	2.14 ± 0.53^*∗∗∗*abb^
SOD (U/g Hb)	1190.6 ± 106	1065.8 ± 160.1^aa^	997.6 ± 109.4^aaa^	1243.7 ± 221^*∗∗∗*bb^
CAT (10^4^ IU/g Hb)	41.5 ± 4.3	59.4 ± 9.7^aaa^	69.9 ± 13.1^aaabb^	49.3 ± 8.3^*∗∗∗*aaabbb^
GPx (U/g Hb)	13.6 ± 1.7	20.9 ± 6.1^aaa^	21 ± 7.8^aaa^	14.2 ± 4.8^*∗∗∗*bbb^
GSH (10^−3^ *μ*mol)	11.1 ± 2.1	10.9 ± 3.8	11.9 ± 3.2	10.6 ± 4.1
CK (IU/L)	15.2 ± 1.6	29.7 ± 2.8^aaa^	30.7 ± 3.5^aaa^	20.3 ± 2.6^*∗∗∗*aaabbb^

The values are expressed as means ± standard deviations (SD) of the means. Statistically significant differences: versus before the surgery: ^*∗∗∗*^ *P* ≤ 0.001; versus Control I: ^a^ *P* ≤ 0.05, ^aa^ *P* ≤ 0.01, and ^aaa^ *P* ≤ 0.001; versus Control II: ^b^ *P* ≤ 0.05, ^bb^ *P* ≤ 0.01, and ^bbb^ *P* ≤ 0.001.

**Table 4:** Statistically significant correlation coefficients between the parameters measured in the patients with cervical spinal cord injury (studied group).

	Parameters	*r*
Before the surgery	TBARS in plasma/TBARS in erythrocytes	0.64^*∗∗∗*^
	CD in plasma/TBARS in plasma	0.78^*∗∗∗*^
	CD in plasma/TBARS in erythrocytes	0.67^*∗∗∗*^
	CD in erythrocytes/TBARS in plasma	0.65^*∗∗∗*^
	CD in erythrocytes/TBARS in erythrocytes	0.71^*∗∗∗*^
	CD in erythrocytes/CD in plasma	0.80^*∗∗∗*^
	CAT/CD in plasma	0.41^*∗∗*^
	CAT/CD in erythrocytes	0.42^*∗∗*^
	GPx/SOD	0.31^*∗*^
	ASIA after hospitalisation/TBARS in plasma	0.67^*∗∗∗*^
	ASIA after hospitalisation/TBARS in erythrocytes	0.60^*∗∗∗*^
	ASIA after hospitalisation/CD in plasma	0.47^*∗∗*^
	ASIA after hospitalisation/CD in erythrocytes	0.46^*∗∗*^
	ASIA 24 h after cranial instrumentation/TBARS in plasma	0.53^*∗∗∗*^
	ASIA 24 h after cranial instrumentation/TBARS in erythrocytes	0.44^*∗∗*^
	ASIA 24 h after cranial instrumentation/CD in erythrocytes	0.35^*∗*^
	ASIA 24 h after cranial instrumentation/GPx	−0.30^*∗*^
	ASIA 24 h after cranial instrumentation/ASIA after hospitalisation	0.80^*∗∗∗*^
	ISS scale/GPx	0.33^*∗*^

7 days after the surgery	CD in plasma/TBARS in plasma	0.47^*∗∗*^
	CD in erythrocytes/TBARS in plasma	0.40^*∗∗*^
	CD in erythrocytes/TBARS in erythrocytes	0.35^*∗*^
	SOD/TBARS in plasma	−0.80^*∗∗∗*^
	SOD/TBARS in erythrocytes	−0.36^*∗*^
	SOD/CD in erythrocytes	−0.51^*∗∗*^
	CAT/TBARS in plasma	0.51^*∗∗∗*^
	CAT/TBARS in erythrocytes	0.64^*∗∗∗*^
	CAT/CD in erythrocytes	0.48^*∗∗∗*^
	CAT/SOD	0.96^*∗∗∗*^
	GPx/TBARS in plasma	0.54^*∗∗∗*^
	GPx/SOD	−0.74^*∗∗∗*^
	GPx/CAT	0.46^*∗∗∗*^
	GSH/CAT	−0.32^*∗*^

^*∗*^ *P* ≤ 0.05; ^*∗∗*^ *P* ≤ 0.01; ^*∗∗∗*^ *P* ≤ 0.001.

TBARS: thiobarbituric acid reactive substances; CD: conjugated dienes; CAT: catalase; GPx: glutathione peroxidase; SOD: superoxide dismutase; GSH: reduced glutathione; ASIA: American Spinal Injury Association Impairment Scale; ISS: Injury Severity Score.
